# Fluency and belief bias in deductive reasoning: new indices for old effects

**DOI:** 10.3389/fpsyg.2014.00631

**Published:** 2014-06-24

**Authors:** Dries Trippas, Simon J. Handley, Michael F. Verde

**Affiliations:** School of Psychology, Cognition Institute, Plymouth UniversityPlymouth, UK

**Keywords:** reasoning, belief bias, signal detection theory, memory, individual differences

## Abstract

Models based on signal detection theory (SDT) have occupied a prominent role in domains such as perception, categorization, and memory. Recent work by Dube et al. (2010) suggests that the framework may also offer important insights in the domain of deductive reasoning. Belief bias in reasoning has traditionally been examined using indices based on raw endorsement rates—indices that critics have claimed are highly problematic. We discuss a new set of SDT indices fit for the investigation belief bias and apply them to new data examining the effect of perceptual disfluency on belief bias in syllogisms. In contrast to the traditional approach, the SDT indices do not violate important statistical assumptions, resulting in a decreased Type 1 error rate. Based on analyses using these novel indices we demonstrate that perceptual disfluency leads to decreased reasoning accuracy, contrary to predictions. Disfluency also appears to eliminate the typical link found between cognitive ability and the effect of beliefs on accuracy. Finally, replicating previous work, we demonstrate that cognitive ability leads to an increase in reasoning accuracy and a decrease in the response bias component of belief bias.

## Introduction

Signal detection theory (SDT) has occupied a prominent role in the study of perception (Green and Swets, 1966), categorization (Ashby and Gott, 1988), memory (Pazzaglia et al., 2013, for a review), and more recently, reasoning (e.g., Rotello and Heit, 2009; Heit and Rotello, 2010). A key feature of SDT is that it provides tools to disentangle response strategies from other cognitive processes by formally specifying the underlying assumptions. SDT assumes that evidence is normally distributed on a strength dimension (e.g., brightness, line length, familiarity, or argument strength), and that the overlap between target and nontarget distributions determines judgment *sensitivity*. As the distance between the means of the distributions increases (i.e., as the distributional overlap gets smaller), sensitivity increases. It is also assumed that a participant will only respond “yes” if the strength of the item under consideration exceeds an internal criterion. This criterion can be shifted independently from sensitivity as a function of task demands or individual preferences. The placement of the criterion determines *response bias*. Certain conditions cause people to adopt a more conservative criterion, meaning that a larger strength value is required to respond “yes,” resulting in a decrease in overall positive responses. Other conditions cause people to adopt a more liberal criterion leading to more “yes” responses overall. The distinction between sensitivity and response bias is a critical one that has not always been adequately addressed by theories of reasoning.

In deductive reasoning, participants are asked to evaluate the logical validity of arguments. A standard belief bias experiment has logic and belief crossed to yield four types of arguments: valid-believable (VB), valid-unbelievable (VU), invalid-believable (IB), and invalid-unbelievable (IU). The dependent measure is how often each item type is endorsed as valid. Traditionally, three indexes are derived from the data:

$$\begin{array}{l} logic\;index=VB+VU-IB-IU\\ belief\;index=VB+IB-VU-IU\\ interaction\;index=VU+IB-VB-IU \end{array}$$

The logic index is interpreted as a measure of reasoning sensitivity. The belief index is typically thought to indicate belief-based response bias. The interaction index is used as a measure of the effect of beliefs on accuracy, that is, enhanced reasoning in the face of unbelievable conclusions.

These interpretations of the effects of logic, belief, and the logic × belief interaction have been called into question by critics who note that the analysis of raw validity judgments relies on unstated assumptions about the nature of evidence that may not be valid (Klauer et al., 2000; Dube et al., 2010). Klauer et al. (2000) pointed out that the interaction index is difficult to interpret for psychometric reasons: changes in proportions starting out from different initial values, as is the case for valid and invalid arguments, cannot readily be compared across conditions. Klauer and colleagues addressed this issue by assuming that argument strength was uniformly distributed, specifying a threshold model as the underlying decision mechanism. Using the multinomial processing tree (MPT) modeling framework, they conducted an impressive series of experiments which culminated in the specification of the selective processing theory of belief bias, according to which beliefs affect both the response stage (response bias) and the reasoning stage (sensitivity, i.e., accuracy). Following up on this work, Dube et al. (2010) pointed out that MPT models and ANOVA of the traditional reasoning indices both predict a linear relationship between the hit rate (responding “valid” to valid problems) and the false alarm rate (responding “valid” to invalid problems). Empirical tests of this assumption, however, demonstrated that it did not hold for reasoning (Dube et al., 2010; see also Trippas et al., 2013, 2014; Heit and Rotello, 2014). Dube et al. put forward SDT as a theoretical framework capable of dealing with this nonlinear relationship for examining how conclusion believability affects reasoning sensitivity and response bias. In applying various SDT models to their own data from syllogistic reasoning tasks, they observed that beliefs did not affect reasoning sensitivity. Instead, the traditional logic × belief interaction was interpreted as originating from a belief driven response bias. This finding was surprising given that most extant theories predict changes in the quality of reasoning (we focus on these theories in more detail in the general discussion). Klauer and Kellen (2011) replied to Dube et al. arguing that MPT threshold models do not necessarily predict linear ROCs. This led them to propose an alternative MPT model capable of fitting curvilinear ROCs (Bröder and Schütz, 2009). Crucially, according to this updated MPT model, there was an effect of believability on both reasoning sensitivity and response bias. Furthermore, the superior fit of this alternative MPT model suggested that conclusions drawn from it were preferable to the SDT model. In turn, Dube et al. (2011) responded to Klauer and Kellen using model recovery simulations to demonstrate that the updated MPT model only fit the data better because it was more flexible than the SDT model, concluding that the SDT model was preferable. In other words, beliefs were thought to affect response bias only.

Following up on this discussion, Trippas et al. (2013) demonstrated that the picture may be more complex. Taking an SDT approach, they observed that while beliefs seemed to only influence response bias under some conditions, changes in sensitivity were evident in other conditions. They pointed to individual differences as an important mediating factor: reasoners with higher levels of cognitive ability were more likely to be influenced by their prior beliefs in the reasoning stage, in contrast to those with lower levels of cognitive ability, who mainly exhibited a belief-based response bias. Although individual differences are also studied in perception (Klein, 1951), categorization (Whitfield, 1984), and memory (e.g., Aminoff et al., 2012; Kantner and Lindsay, 2012), they have played a particularly important role in the development of reasoning theories (see Stanovich and West, 2000, 2008, for reviews), suggesting that it is perhaps unsurprising that ignoring them has led to conflicting findings.

The fine-grained conclusions drawn by Trippas et al. (2013) could not have been confidently reached without the use of a formal modeling procedure such as SDT. In fact, recent work by Heit and Rotello (2014) combined statistical simulations with an experimental approach to underscore that the issue extends even beyond the interaction index. According to their data, all traditional belief bias indices are problematic, with simulations showing that they lead to an (unacceptably) inflated Type 1 error rate—and thus unreliable conclusions. In this paper, we follow up on Heit and Rotello's work by describing three SDT indices which are designed to disentangle sensitivity and response bias, by explicitly estimating the various parameters of the underlying distributions of argument strength for each participant. In the final part of this paper, we then apply these indices to a case study in which the examination of individual differences (cognitive ability) is illuminating: the role of perceptual fluency on belief bias in syllogistic reasoning.

### SDT indices

SDT indices for data resulting from binary decisions (e.g., valid/invalid) are *d*' and *c*. *d*' is a measure of sensitivity, representing the distance between the nontarget (i.e., invalid) and target (i.e., valid) distributions in units of standard deviations. *c* is a measure of criterion placement, with lower values indicating a more liberal response bias (i.e., more yes responses).

$$\begin{array}{l} d^{\prime }=z\left(H\right)-z\left(F\right)\\ c=-\frac{z\left(H\right)+z\left(F\right)}{2} \end{array}$$

With *H* = *p*(“valid” | valid) and *F* =p(“valid” | invalid).

A problem with *d*' and *c* is that they entail the assumption of equal variance between the target (valid) and nontarget (invalid) distributions, an assumption that is often violated. Alternative indices which do not require the equal variance assumption are *d*_*a*_ and *c*_*a*_ (e.g., Macmillan and Creelman, 2005):

$$\begin{array}{l} d_{a}=\sqrt{\left(\frac{2}{1+s^{2}}\right)}*\left[z\left(H\right)-s*z(F)\right]\\ c_{a}=\frac{-\sqrt{2}*s}{\sqrt{\left(1+s^{2}\right)}*(1+s)}*[z\left(H\right)\;+z(F)] \end{array}$$

**Figure 1:**
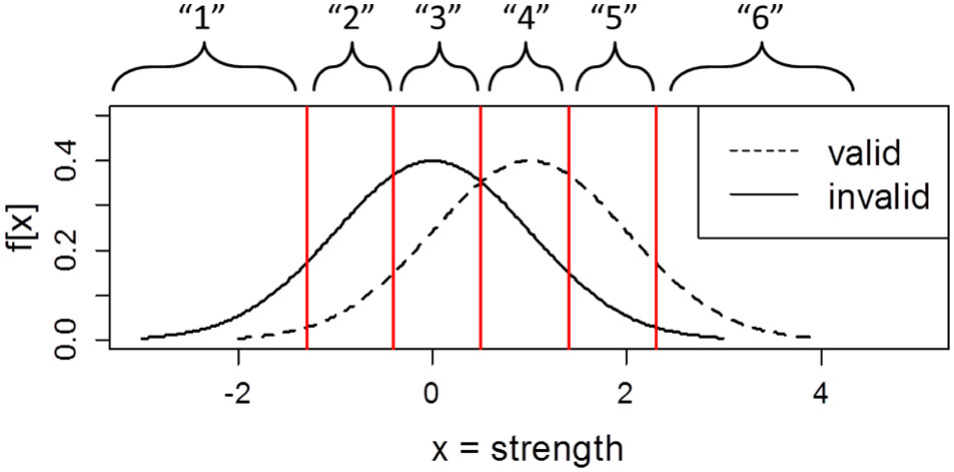
**Argument strength distributions of valid and invalid problems demonstrating the link between confidence ratings and response criteria**.

In order to calculate *d_a_* and *c_a_*, one needs to estimate *s*, which represents the ratio of the variance of the noise (nontarget) and signal (target) distributions (also referred to as the z-ROC slope). *s* can be estimated using the receiving operator characteristic (ROC) procedure. In the ROC procedure, participants may be instructed to supplement each binary decision with a confidence rating. Combining the binary decisions and the three-point confidence scale yields six response classes (Figure 1).

$$\begin{array}{l} 6=yes+3\;(high\;confidence\;valid\;response)\\ 5=yes+2\;(moderate\;confidence\;valid\;response)\\ 4=yes+1\;(low\;confidence\;valid\;response)\\ 3=no+1\;(low\;confidence\;invalid\;response)\\ 2=no+2\;(moderate\;confidence\;invalid\;response)\\ 1=no+3\;(high\;confidence\;invalid\;response) \end{array}$$

An ROC curve plots hits against false alarms at each confidence level. *s* can be estimated from the slope of the z-transformed ROC^1^. Using *d_a_* and *c_a_*, one can turn to a set of indices that are analogous to the traditional indices used to study belief bias but which are better justified given the empirically observed nature of evidence distributions (Dube et al., 2010, 2011; Heit and Rotello, 2014; but see also Klauer and Kellen, 2011; Singmann and Kellen, 2014). Note that to use the following formulae, *s* needs to be estimate three times: once for the full ROC collapsed across believability, once for the believable condition, and once for the unbelievable condition:

$$\begin{array}{l} SDT\;logic\;index=\;d_{a}\\ SDT\;belief\;index=\;c_{a}unbelievable-\;c_{a}believable\\ SDT\;interaction\;index=\;d_{a}unbelievable-\;d_{a}believable \end{array}$$

The SDT-logic index measures overall reasoning sensitivity. The SDT-belief index is the relative difference in response bias between the unbelievable and the believable condition. Higher values indicate a greater tendency to accept believable problems. Finally, the SDT-interaction index indicates the sensitivity difference between believable and unbelievable arguments, or the belief-accuracy effect. Much of the debate on the nature of belief bias in reasoning has focused on the nature of this logic × belief interaction. We return to this point in the general discussion.

### Summary

An increasing body of evidence (Klauer et al., 2000; Dube et al., 2010, 2011; Klauer and Kellen, 2011; Trippas et al., 2013, 2014; Heit and Rotello, 2014) suggests that the use of traditional analysis techniques for the study of reasoning should be avoided, as these techniques are very likely to lead to inappropriate conclusions about the nature of belief bias. Simulations have shown that using the traditional indices puts researchers at risk of inflated Type 1 error rates, something which can be avoided by applying formal modeling techniques such as SDT (Heit and Rotello, 2014). Using formal modeling procedures for the study of belief bias can be impractical, however, because the use of more advanced experimental designs may entail the fit and comparison of models with an untenably large number of parameters. In contrast, the SDT indices approach described here reconciles the SDT approach with the more classical approach previously offered by the traditional belief bias indices.

Using the SDT-indices method is straightforward. First, when conducting a standard belief bias experiment, ensure to collect confidence ratings alongside each binary validity judgment. Note that this does necessitate the use of a three-point scale: estimates can always be recoded and collapsed afterwards. Second, combine the validity judgments and the confidence ratings into the required number of bins (six in our case) and calculate conditional frequencies denoting how often each response is made per condition and participant [i.e., *f*(6 | valid), *f*(5 | valid),…, *f*(1 | valid), repeat for invalid]. Third, use one of the available tools^1^ to fit the unequal variance SDT (UVSDT) model to each participant's counts to estimate *s _total_, s _believable_*, and *s _unbelievable_*. Occasionally, for some participants, the model will not produce a reliable fit. In this case it is advised to simply assume *s* = 1 in which case the measures generalize to their equal variance SDT counterparts *d*′ and *c*. Alternatively, an average of *s* across all other participants can be used. Using the formulae outlined above, calculate *d_a total_, c_a total_, d_a believable_, c_a believable_, d_a unbelievable_*, and *c_a unbelievable_* for each participant. Finally, using these estimates, the SDT-logic, SDT-belief, and SDT-interaction indices can be calculated for analysis using standard procedures such as ANOVA. We now demonstrate the use of this technique by investigating the role of perceptual disfluency on belief bias in syllogistic reasoning.

## Fluency and belief bias

Fluency, the ease of processing a stimulus, has been a focus of interest in a variety of cognitive domains. In memory, fluency has been shown to influence both response bias and sensitivity. Some fluency-related memory illusions seem to be pure bias effects (e.g., Verde et al., 2010). On the other hand, perceptual disfluency (i.e., visual degradation) at encoding can produce better long-term recognition (e.g., Mulligan, 1996), a counterintuitive effect that has been linked to the enhanced effort needed to encode difficult-to-perceive materials. A similar effect of disfluency has been reported in reasoning. Alter et al. (2007) found that presenting reasoning problems in difficult to read font led to more accurate judgments. However, their finding has been difficult to replicate (Thompson et al., 2013). As Dube et al. (2010) noted in the study of belief bias, apparent inconsistencies in results can sometimes be traced to the use of analytic techniques that fail to appropriately distinguish between response bias and sensitivity. The studies of Alter et al. and Thompson et al. measured accuracy by analysing proportion correct, equivalent to the use of the traditional reasoning indices. They also exclusively focused on the effect of accuracy, ignoring any potential effects of fluency on response bias. This motivated our examination of the effect of disfluency on belief bias in syllogistic reasoning using the alternative SDT indices.

### Methods

#### Participants

Seventy-six undergraduate psychology students from Plymouth University (UK) participated in exchange for course credit. The experiment was approved by the ethical committee of the Faculty of Science and Environment at Plymouth University.

#### Design

Logic (valid vs. invalid) and conclusion believability (believable vs. unbelievable) were manipulated within subjects. Perceptual fluency (fluent: standard, easy to read font, *n* = 38 vs. disfluent: difficult to read font, *n* = 38) was manipulated between subjects.

#### Materials

Following Trippas et al. (2013), a unique list of problems was created for each participant by randomly assigning item contents to complex logical structures for each participant anew (for a list of the structures see the appendix of Dube et al., 2010). Each list contained 64 syllogisms, containing equal numbers of VB, VU, IB, and IU arguments.

In the fluent condition, arguments were presented on a 1080p LCD monitor in an easy to read font (Courier New, 18 pt., bold, black on white background). In the disfluent condition, arguments were presented in a difficult to read font (Brush Script MT, 15 pt., italic, light gray on white background, cf. Thompson et al., 2013).

We measured cognitive ability using the short form of Raven's advanced progressive matrices (APM-SF) which has a maximum score of 12 (Arthur and Day, 1994). This is a sound instrument for assessing fluid cognitive ability in a short time frame (Chiesi et al., 2012).

#### Procedure

Participants were tested on individual computers in small groups no larger than five. After signing a consent form they were presented with standard deductive reasoning instructions stating:

*In this experiment, we are interested in people's reasoning. For each question, you will be given some information that you should assume to be true. This will appear ABOVE a line. Then you will be asked about a conclusion sentence BELOW the line. If you judge that the conclusion necessarily follows from the premises, you should answer “Valid,” otherwise you should answer “Invalid.” After each validity judgment, you will be asked how confident you are in this judgment*.

$$\begin{array}{l} 1=you\;are\;not\;confident\;at\;all\\ 2=you\;are\;moderately\;confident\\ 3=you\;are\;very\;confident \end{array}$$

*Please try to make use of all three confidence response categories*.

After four practice trials (one of each item type), participants were presented with the 64 reasoning problems. Upon completion of the reasoning task, the APM-SF was administered, after which participants were debriefed.

### Results and discussion

#### SDT-indices analysis

To investigate the impact of perceptual disfluency on reasoning sensitivity, belief bias, and the effect of beliefs on accuracy, we calculated the SDT-logic, SDT-belief, and SDT-interaction indices. For each participant, we fit the UVSDT model with seven parameters (five criteria, one mean, and one standard deviation, hereafter, *s*) in three different ways. For the SDT-logic index, the model was fit to the ROC collapsed across believability to provide an overall estimate of *s _total_*, allowing us to calculate *d_a_*. Next, we fit the same model separately for the believable condition and the unbelievable condition to estimate *s _believable_* and *s _unbelievable_*. Based on these fits, *c_a believable_, c_a unbelievable_, d_a believable_*, and *d_a unbelievable_* were calculated. In turn, these values were used to calculate the SDT-belief and SDT-interaction indices using the formulas outlined above.

#### Model fit

We inspected absolute model fits in terms of *G*^2^ to ensure that our analyses are not affected by artifacts produced by ill-fitting models. Across all 228 (3 models × 76 participants) fits, there were 12 cases in which the model could not be fit because the participant did not employ a sufficient number of response options. For these participants we calculated the statistics assuming a z-ROC slope *s* of 1 (i.e., *d*' and *c*). There were only 8 cases (<4%) for which the model did not fit the data well (i.e., *p* < 0.05). The model provided a good fit of the data for over 96% of the cases.

#### Preliminary analysis

We compared the three indices with 0 using a one sample *t*-test to investigate whether participants reasoned above chance, whether they showed the belief bias, and whether beliefs affected accuracy (see, for instance, Evans and Curtis-Holmes, 2005, for a similar approach using traditional belief bias indices). All *t*-tests and ANOVAs are supplemented with Bayes factors (BF) in terms of evidence in favor of alternative hypothesis/evidence in favor of the null hypothesis using the JZS prior method using default scaling (Rouder et al., 2009, 2012; Bayes factors were calculated using the “BayesFactor” package in R, MCMC resampling was used for the ANOVAs). Note that *BF* > 1 indicates evidence in favor of the alternative hypothesis, with *BF* > 3 indicating substantial evidence, *BF* > 10 indicating strong evidence, and *BF* > 100 indicating conclusive evidence. *BF* < 1 indicates evidence in favor of the null hypothesis, with <0.3, <0.1, and <0.01 indicating substantial, strong, and conclusive evidence in favor of the null, respectively.

Participants performed well above chance, *t*(75) = 8.41, *p* < 0.001, *BF* > 1000. They also showed the standard belief bias, *t*(75) = 6.72, *p* < 0.001, *BF* > 1000. Finally, beliefs affected reasoning accuracy, *t*(75) = 2.81, *p* = 0.006, *BF* = 4.78.

#### Main analysis

To test the main prediction that disfluency affects reasoning accuracy, we performed a One-Way ANOVA with the SDT-logic index as the dependent variable and condition (disfluent vs. fluent) as a between subjects factor. Fluency affected accuracy, *F*(1, 74) = 6.22, *p* = 0.015, ω^2^ = 0.078, *BF* = 3.30. Contrary to predictions, however, accuracy was *lower* in the disfluent (*d_a_* = 0.51, *Az* = 64%) compared to the fluent condition (*d_a_* = 0.92, *Az* = 74%).

To investigate whether fluency affected response bias, we analyzed the SDT-belief index using a One-Way ANOVA, with condition (disfluent vs. fluent) as a between subjects factor. Fluency did not impact on belief-based response bias, *F*(1, 74) < 1, *p* = 0.36, *BF* = 0.34.

Finally, we investigated whether fluency affected the belief-accuracy effect by analysing the SDT-interaction index. It did not, *F*(1, 74) < 1, *p* = 0.69, *BF* = 0.25. Means and standard errors can be found in Table 1.

**Table 1:** **Reasoning accuracy and criterion placement per condition**.

**Ability**	**Condition**	***d_a_***	***c_a_ bel***	***c_a_ unbel***	***d_a_ bel***	***d_a_ unbel***
Total	Collapsed	0.71 (0.08)	−0.62 (0.07)	0.14 (0.08)	0.57 (0.07)	0.76 (0.10)
	Fluent	0.92 (0.11)	−0.55 (0.08)	0.11 (0.11)	0.75 (0.09)	0.97 (0.14)
	Disfluent	0.51 (0.12)	−0.70 (0.11)	0.17 (0.11)	0.39 (0.11)	0.56 (0.13)
Higher	Collapsed	1.06 (0.14)	−0.41 (0.11)	0.01 (0.11)	0.86 (0.10)	1.09 (0.16)
	Fluent	1.35 (0.17)	−0.21 (0.09)	−0.07 (0.13)	1.00 (0.14)	1.48 (0.19)
	Disfluent	0.81 (0.19)	−0.57 (0.17)	0.07 (0.17)	0.74 (0.14)	0.76 (0.22)
Lower	Collapsed	0.42 (0.08)	−0.80 (0.08)	0.26 (0.11)	0.32 (0.09)	0.49 (0.11)
	Fluent	0.60 (0.11)	−0.79 (0.10)	0.24 (0.15)	0.56 (0.11)	0.60 (0.17)
	Disfluent	0.21 (0.11)	−0.82 (0.15)	0.27 (0.15)	0.04 (0.13)	0.35 (0.13)

*Means (standard errors). Bel = believable; unbel = unbelievable. Descriptive statistics are shown for all participants (total) as well as subgroups of higher and lower cognitive ability separately*.

#### Individual differences analysis

To ensure that our random assignment was successful, we compared cognitive ability between conditions using a two samples *t*-test. Both fluency groups were matched on cognitive ability: *t*(74) < 1, *p* = 0.48, *BF* = 0.29.

To test whether the effect of fluency on accuracy was moderated by cognitive ability, we performed a 2 (condition: fluent vs. disfluent) × 2 (cognitive ability: higher vs. lower, based on median splits) between subjects ANOVA on the SDT-logic index. There was a main effect of cognitive ability, *F*(1, 72) = 21.01, *p* < 0.001, ω^2^ = 0.23, *BF* > 1000, indicating that reasoning accuracy was higher for the higher ability subgroup. The main effect of fluency remained significant, *F*(1, 72) = 9.96, *p* = 0.002, ω^2^ = 0.12, *BF* = 15, confirming that fluency decreased reasoning accuracy, even when variance due to individual differences was accounted for. There was no interaction between cognitive ability and fluency, *F*(1, 72) = 0.25, *p* = 0.62, *BF* = 0.35.

To investigate the role of cognitive ability and fluency on the response bias component of belief bias, we conducted a 2 (cognitive ability) × 2 (fluency) between subjects ANOVA on the SDT-belief index. A main effect of cognitive ability indicated that higher ability people were less likely to show the standard belief bias, *F*(1, 72) = 9.59, *p* = 0.003, ω^2^ = 0.12, *BF* = 13.96. There was no main effect of fluency, *F*(1, 72) = 1.48, *p* = 0.23, *BF* = 0.47. The interaction between ability and fluency was also not significant, *F*(1, 72) = 1.07, *p* = 0.31, *BF* = 0.47.

Finally, we also analyzed the SDT-interaction index using a 2 (cognitive ability) × 2 (fluency) between subjects ANOVA. There was no main effect of cognitive ability, *F*(1, 72) < 1, *p* = 0.63, *BF* = 0.26, or fluency, *F*(1, 72) < 1, *p* = 0.65, *BF* = 0.29. The analysis did reveal that there was a significant ability × fluency interaction, *F*(1, 72) = 7.03, *p* = 0.01, ω^2^ = 0.09, *BF* = 5.04, suggesting that the effect of cognitive ability on the belief-accuracy effect was mediated by fluency. Follow up tests comparing the higher and lower ability groups revealed the following pattern. For the lower cognitive ability participants, there was no effect of fluency, *t*(39) = −1.44, *p* = 0.15, *BF* = 0.70. For the higher ability group, the SDT-interaction index was higher for the fluent compared to the disfluent condition, *t*(33) = 2.28, *p* = 0.028, *BF* = 2.29. Follow-up tests produced strong evidence for an effect of beliefs on accuracy for the higher ability subgroup in the fluent condition, *t*(15) = 3.37, *p* = 0.004, *BF* = 11.34. For the lower ability subgroup in the fluent condition, beliefs did not affect reasoning accuracy, *t*(21) = 0.28, *p* = 0.78, *BF* = 0.23.

## General discussion

Although SDT has taken a prominent role in the development of theoretical accounts in domains such as perception, categorization, and memory, its application to reasoning has been a fairly recent development (e.g., Heit and Rotello, 2005). As has been previously argued in the context of belief bias, failure to specify assumptions about the nature of evidence can lead to interpretations of data that are misleading or incorrect (Klauer et al., 2000; Dube et al., 2010). The benefit of a formal model like SDT is in its specification of assumptions. The provision of analytic tools that allow the separation of sensitivity and response bias is an added advantage when it comes to the study of phenomena like fluency effects, which are known (in the domain of memory) to potentially impact both. Our findings from the manipulation of fluency in a reasoning task differed from those of two previous studies: we found that disfluency led to a reduction in reasoning sensitivity, in contrast to the improvement reported by Alter et al. (2007) and the null effect reported by Thompson et al. (2013). While we can only speculate about the extent to which differences in measurement tools may have contributed to inconsistent findings, it is important that the SDT indices used here do not suffer from the theoretical shortcomings noted with traditional indices. For the current study, the main advantage of the SDT-indices was a decreased probability of Type 1 errors, leading to increased confidence in our findings compared to previous research where the conclusions are drawn on the basis of more traditional analysis techniques.

Perceptual fluency mediated the link between cognitive ability and the belief-accuracy effect. When arguments were presented in a perceptually disfluent fashion, the link between cognitive ability and motivated reasoning disappeared. It may be that increased difficulty discourages or distracts from the use of what is essentially a higher-level strategy. On a similar note, Trippas et al. (2013) found that time pressure reduced both overall reasoning sensitivity and the belief-accuracy effect among higher ability participants. Drawing this comparison, it may be that a more parsimonious interpretation of the role of disfluency in reasoning is simply the following: making a task more difficult hogs the necessary resources typically used to afford an accuracy advantage in the face of unbelievable arguments.

While we failed to replicate the disfluency advantage, we did replicate other findings with respect to belief bias. Trippas et al. (2013) observed a logic × belief interaction effect, mediated by cognitive ability, that is usually interpreted as a belief-accuracy effect. Certain theories of belief bias (e.g., mental models theory: Oakhill et al., 1989; selective processing theory: Klauer et al., 2000; Evans et al., 2001; modified selective processing theory: Stupple et al., 2011; dual process theory (DPT): Evans, 2007) predict that the accuracy effect stems from a type of “motivated reasoning,” whereby the unbelievable nature of a conclusion triggers more effortful reasoning, leading to increased normative performance. Other theories (e.g., misinterpreted necessity: Evans et al., 1983; metacognitive uncertainty: Quayle and Ball, 2000; verbal reasoning theory: Polk and Newell, 1995; modified verbal reasoning theory: Thompson et al., 2003) predict that the interaction stems from an asymmetrical belief-based response bias for valid and invalid arguments, driven by various metacognitive backup strategies. These strategies are thought to be engaged when reasoning becomes too complicated. Finally, some theories (the response bias-only account: Dube et al., 2010; the probability heuristics model: Chater and Oaksford, 1999) do not predict a logic × belief interaction, although there may be ways to reconcile our findings with these theories. Pure response bias may hold under some conditions but not others (Heit and Rotello, 2014). The probability heuristics model might be extended to allow for a reasoning advantage in the face of unbelievable problems by assuming that believability influences the probability of using the p-entailment heuristic. Of the various theories just described, only three make explicit predictions about the role of individual differences in belief bias.

According to DPT, motivated reasoning requires working memory capacity (WMC), in contrast to response bias, which is a by-product of effortless heuristic processing (Evans, 2007). The finding that higher cognitive ability (a correlate of WMC) leads to increased motivated reasoning and decreased response bias is consistent with this theory. Modified selective processing theory, an algorithmic level reasoning theory part of the DPT framework similarly predicts that reasoning ability is linked to an increase in motivated reasoning and a decrease in response bias. One notable addition is the prediction of a curvilinear relationship between motivated reasoning and cognitive capacity: excellent reasoners who are near ceiling necessarily resist the influence of their prior beliefs, leading to perfect normative performance, thus showing no difference in accuracy as a function of believability. Metacognitive uncertainty, finally, predicts that those of *lower* WMC are more inclined to show the logic × belief interaction. The current findings run counter to this prediction.

How can future research further distinguish between these reasoning theories? Many critics would argue that the study of belief bias has focused on traditional or classical syllogisms for too long, ignoring other forms of reasoning more prevalent in daily life. The study of belief bias in statistical syllogisms, reasoning problems which include the quantifiers “Most” and “Few” alongside the traditional ones, will provide a fruitful direction. All of the belief bias theories discussed here evaluate reasoning competence with relation to the norm of classical logic. However, according to this framework, statistical syllogisms are invalid. If future experiments were to demonstrate accuracy changes as a function of beliefs and/or fluency for traditional and statistical syllogisms in equal measures, then this would suggest that traditional belief bias theories only capable of dealing with classical logic must be incorrect in their current form.

### Conflict of interest statement

The authors declare that the research was conducted in the absence of any commercial or financial relationships that could be construed as a potential conflict of interest.

## Footnotes

^1^An excel based tutorial containing a fitting algorithm is available online: http://www.psych.umass.edu/download.php?site=48&File=SDT_Tutorial.zip&type=application/x-zip-compressed. A second option is to use the R-package MPTinR, which can also be used to fit SDT models (Singmann and Kellen, 2013). A third option is to use Systat or SPSS to fit the SDT model (see DeCarlo, 2003, for a tutorial using the latter). Finally, a more straightforward if less reliable method is to estimate the slope of the z-ROC using linear regression. First, estimate the cumulative proportions for the valid and the invalid problems, i.e., *p*(6 | valid), *p*(6 + 5 | valid), …, *p*(6 + 5 + … + 2 | valid), and the same for the invalid problems. Next, calculate the z-score of each observation (that is, a transformation using the quantile function of the standard normal distribution). Finally, regress the z-transformed hits against the z-transformed false alarms for an estimate of the z-ROC slope *s*.
